# Coffee Decreases the Risk of Endometrial Cancer: A Dose–Response Meta-Analysis of Prospective Cohort Studies

**DOI:** 10.3390/nu9111223

**Published:** 2017-11-09

**Authors:** Alessandra Lafranconi, Agnieszka Micek, Fabio Galvano, Sabrina Rossetti, Lino Del Pup, Massimiliano Berretta, Gaetano Facchini

**Affiliations:** 1 The Research Centre on Public Health, University Milano-Bicocca, via Pergolesi 33, 20900 Monza, Italy; alessandra.lafranconi@unimib.it; 2 Department of International Health, FHML, CAPHRI, Maastricht University, 6229 Maastricht, The Netherlands; 3 Department of Epidemiology and Population Studies, Jagiellonian University Medical College, 31008 Krakow, Poland; agnieszka.micek@uj.edu.pl; 4 Department of Biomedical and Biotechnological Sciences, University of Catania, 95124 Catania, Italy; fgalvano@unict.it; 5 Division of Medical Oncology, Department of Uro-Gynaecological Oncology, Istituto Nazionale Tumori ‘Fondazione G. Pascale’—IRCCS, 80131 Naples, Italy; sabrinarossetti@virgilio.it; 6 Gynecological Oncology, National Cancer Institute—IRCCS, 33081 Aviano (PN), Italy; ldelpup@cro.it; 7 Department of Medical Oncology, National Cancer Institute—IRCCS, 33081 Aviano (PN), Italy; mberretta@cro.it

**Keywords:** coffee, caffeine, postmenopausal, endometrial cancer, prospective cohort, meta-analysis

## Abstract

Aim: The aim of this study was to perform a comprehensive meta-analysis of the association between coffee consumption and risk of endometrial cancer. Methods: Eligible studies were identified by searching the PubMed and EMBASE databases. The dose–response relationship as well as the risk of endometrial cancer for the highest versus the lowest categories of coffee consumption were assessed. Subgroup analyses considering the menopausal and receptor statuses, the smoking status, and the BMI (Body Mass Index) were performed in order to identify potential confounders. Results: We identified a total of 12 studies eligible for meta-analysis. A dose–response meta-analysis showed a decreased risk of endometrial cancer. Moreover, a subgroup analysis indicated that coffee consumption is significantly associated with a decreased risk of postmenopausal cancer. Increasing coffee consumption by four cups per day was associated with a 20% reduction in endometrial cancer risk (relative risk (RR) 0.80; 95% confidence interval (CI) 0.72 to 0.89) and with a 24% reduction in postmenopausal cancer risk (RR 0.76; 95% CI 0.69 to 0.83). Conclusions: Our findings suggest that increased coffee consumption is associated with decreased risk of endometrial cancer, and this association is observed also for postmenopausal cancer.

## 1. Introduction

Endometrial cancer is the third most common female cancer, after breast cancer and cervical cancer: in 2012, worldwide, there were over 1.2 million women with a diagnosis of endometrial cancer made in the previous 5 years. Similarly, in Europe there were 370,000 women with the same diagnosis [1]. The burden of non-communicable diseases, including cancers, has been associated with dietary habits, such as the consumption of fibers, sugar, saturated fatty acids, and trans fatty acids [2,3,4]. Among others, coffee consumption has also been shown to potentially affect human health [5]. Moderate coffee consumption has been suggested to improve metabolic health and to decrease the risk of mortality [6,7]. Regarding cancer risk, coffee consumption has been associated with decreased risk of liver [8], prostate [9], pancreatic [10], and colon cancer [11], suggesting a potential role of coffee in cancer prevention. Coffee is composed of a variety of compounds, including polyphenols, diterpenes and melanoidins, that have been reported to modulate anti-inflammatory and anti-oxidant body responses, which may explain the potential beneficial effects of coffee in cancer prevention [12,13].

A decreased risk of endometrial cancer in women who regularly drink coffee has been documented in retrospective and prospective studies: Je and Giovannucci performed a meta-analysis including 10 case-control and 6 cohort studies and found a significant inverse association of endometrial cancer with coffee consumption in the highest versus the lowest category of coffee intake [14]. The dose–response analysis showed a decrease of 8% in the risk of endometrial cancer for an increment of one cup of coffee per day [14]. Similar conclusions were reported by Zhou and colleagues in a meta-analysis of prospective studies: they found a significant reduction of endometrial cancer risk associated with increased coffee consumption, with a linear dose–response relationship [15]. Significant results were obtained also in the meta-analysis of Yang and colleagues, who estimated a decreased risk of endometrial cancer of about 10% for every additional daily cup of coffee [16]. Results are promising but evidence is not yet conclusive, especially regarding specific population subgroups (e.g., in relation to body mass index (BMI), age, geographic place of residence, and ethnicity); in light of the high worldwide consumption of coffee, further research is needed [17,18]. Moreover, new cohort studies have been published, and some overlapping cohorts were not considered in the previous meta-analyses [19]. Thus, the aim of this study was to update the current evidence on the association between coffee intake and risk of endometrial cancer, providing insights on potential effect modifiers or confounding factors, including the BMI and the menopausal status.

## 2. Methods

We followed the Meta-Analysis of Observational Studies in Epidemiology (MOOSE) protocols throughout the design, execution, analysis, and reporting of the current meta-analysis (Supplementary Table S1) [20].

### 2.1. Search Strategy

We conducted a comprehensive literature search using two medical databases, namely, PubMed (http://www.ncbi.nlm.nih.gov/pubmed/) and EMBASE (http://www.embase.com/); the databases were screened from the earliest available online indexing year up to March 2017, with English-language restriction. We included the following search terms: *(coffee OR caffeine OR beverages OR diet Or dietary) AND (endometrial OR endometrium) AND (cancer OR carcinoma OR neoplasm)* (Supplementary Table S2). Two authors evaluated independently the pertinence of the retrieved studies and obtained full texts of the relevant ones. We limited our search to prospective cohort studies that evaluated the association between dietary coffee intake and the risk of endometrial cancer in the overall female population. Studies were included if they provided any of the following risk estimates: relative risks (RRs) or hazard ratios (HRs). We excluded studies that reported insufficient statistics or insufficient coffee consumption categories (less than three). Among the included manuscripts, all references were also examined in order to maximize the number of relevant studies through the addition of studies not previously identified. When duplicate publications from the same study were identified, we chose the report that provided the largest number of cases (or the entire cohort), or the longest follow-up for each endpoint of interest. Full texts of potentially relevant articles were assessed independently for eligibility by two different authors.

### 2.2. Data Extraction

A standardized extraction form was used to extract data from each study included in the meta-analysis. The following information was collected: (1) first author name; (2) year of publication; (3) study cohort name; (4) country; (5) number of participants; (6) sex of participants; (7) age range of the study population at baseline; (8) categories of consumption; (9) follow-up period; (10) endpoints and cases; (11) distributions of cases and person-years, RRs, HRs, and 95% CIs (confidence interval) for all categories of exposure; (12) covariates used in adjustments. Two authors independently performed such process, and discrepancies were discussed and resolved by consensus. Each study was critically appraised through the use of a quality scale, namely, the Newcastle-Ottawa Quality Assessment Scale [21], which consists of three variables of quality as follows: selection (4 points), comparability (2 points), and outcome (3 points), for a total score of 9 points (9 representing the highest quality).

### 2.3. Statistical Analysis

In this meta-analysis, HRs were deemed equivalent to relative risks (RRs) [22]. For each study, extracted statistical estimates were RRs and HRs with 95% CI for all categories of exposure. Random-effects models were used to calculate pooled RRs with 95% CI for the highest versus the lowest categories of exposure (such analysis aimed to assess the presence of a relationship between coffee intake and risk of endometrial cancer). In performing such analysis, for each study, we used the risk estimate derived from the most fully adjusted models (analysis of the pooled RR). Heterogeneity was assessed using the Q test and *I*^2^ statistic. The level of significance was set equal to 0.10 for the Q test. The *I*^2^ statistic explains the amount of total variation that could be attributed to heterogeneity. *I*^2^ values ≤25%, 25–50%, 50–75%, and >75% indicated no, small, moderate, and significant heterogeneity, respectively. To evaluate the stability of the results and potential sources of heterogeneity, a sensitivity analysis by exclusion of one study at a time was performed. Moreover, a subgroup analysis was performed in order to check for potential sources of heterogeneity according to the geographical area. To test for potential confounders and effect modifiers, other subgroup analyses were performed (by menopausal status, receptor status, BMI, smoking status, and coffee type). The publication bias was evaluated by a visual investigation of the funnel plots for potential asymmetry.

To better explore the relationship between exposure and outcome, a dose–response meta-analysis was performed (a dose–response analysis was preferred over other designs, e.g., meta-regression, to better compare our results with previous studies and to better draft potential recommendations whether evidence would support the present findings). Extracted data were stratified by the level of coffee intake, and distributions of cases and person-years (when available), and RRs or HRs with 95% CIs for ≥3 exposure categories were included. The median or mean intake of coffee in each category was assigned to the corresponding RR or HR with the 95% CI for each study. If coffee consumption was reported in a range of intake, we used the midpoint of the range. Similarly, when the highest category was open-ended, we assumed that the width of the category would be the same as the adjacent category. In the case of an open-ended lowest category, we clearly set the lower boundary to zero. Two-stage random-effects dose–response meta-analysis was performed to examine linear and non-linear relationship between coffee intake and risk of endometrial cancer. In the first stage, the method of Greenland and Longnecker (generalized least-squares, GLS) was used to calculate study-specific coefficients on the basis of results across categories of coffee intake, taking into account the correlation within each set of retrieved RRs and HRs [23,24]. Through the use of restricted cubic splines with three knots at fixed percentiles (25%, 50%, and 75%) of the distributions, non-linear dose–response analyses were modelled [25], and the coefficients (that had been estimated within each study by performing random-effects meta-analysis) were combined. To estimate the relative risks, we used the method of DerSimonian and Laird in linear dose–response meta-analyses, and the multivariate extension of the method of moments in non-linear dose–response meta-analyses. We calculated a *p*-value for non-linearity by testing the coefficient of the second spline as equal to zero. We performed all analyses with R software version 3.0.3 (Development Core Team, Vienna, Austria).

## 3. Results

### 3.1. Study Characteristics

**Figure 1:**
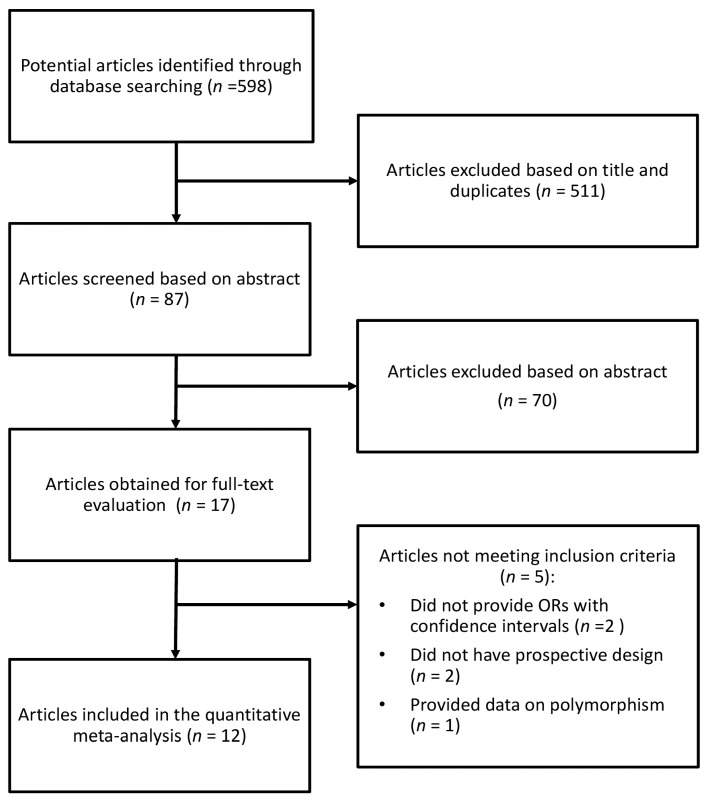
Selection process of relevant studies reporting on the association between coffee consumption and endometrial cancer risk.

The search identified 598 studies, of which 511 were excluded after reviewing the title, and 70 on the basis of the abstract (Figure 1). Of the 17 publications selected, five were excluded for the following reasons: (1) the article did not provide risk measurements with confidence intervals; (2) the article did not have a prospective design; (3) the article provided data only on genetic polymorphism. For the analysis on the association between coffee consumption and endometrial cancer risk, 12 studies were eligible [16,26,27,28,29,30,31,32,33,34,35,36]. Two cohorts, NOWAC and VIP [27,33], were excluded from the main analysis because part of theirs cases are included in the multicenter study EPIC [32]. However, an alternative analysis was performed by including these cohorts and excluding the EPIC study. One article was used only for subgroup analysis [31]. Studies eligible for the main analysis comprised 1,404,541 participants and 10,548 endometrial cancer cases. The main characteristics of the studies included in the meta-analysis are described in Table 1. Seven studies provided relative risk measurements for the postmenopausal [26,27,28,29,30,31,35] status, and two for the premenopausal status [27,31]. Five studies were conducted in the USA [28,29,30,31,35], five in Europe [16,26,27,33,36], one in Asia [34], and one on a cohort from Europe and North America [32]. The follow-up in prospective cohort studies ranged from about 6 to 26 years, and the age range at study baseline was between 25 and 74 years (with almost all studies covering the age range between 40 and 60 years).

### 3.2. Summary Relative Risk for Highest vs. Lowest Category of Coffee Consumption

**Figure 2:**
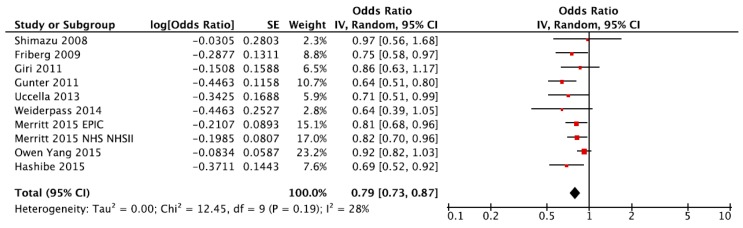
Forest plot of summary relative risks (RRs) of endometrial cancer for the highest versus the lowest (reference) category of coffee consumption. Exposure categories are reported as identified in the original studies; in the dose–response analysis they were harmonized (range: 0–9 cups).

The summary Relative Risk (RR) of endometrial cancer for the highest versus the lowest category of coffee consumption was RR = 0.79, 95% CI: 0.73, 0.87, with small heterogeneity *I*^2^ = 28%, *p* = 0.19, (Figure 2); no publication bias was found after a visual inspection of the funnel plot (Supplementary Figure S1). When we performed the alternative analysis that included the NOWAC and VIP cohorts instead of the EPIC study (used in the main analysis), the RR was even lower and equal to 0.73 (95% CI: 0.64, 0.84), with moderate heterogeneity (*I*^2^ = 44%, *p* = 0.0375).

The associations for caffeinated and decaffeinated coffee were consistent with the aforementioned relationship (RR = 0.65, 95% CI: 0.50, 0.85 for caffeinated coffee, and RR = 0.76, 95% CI: 0.62, 0.93 for decaffeinated coffee, Table 2).

When taking into account the menopausal status, a significant decrease in the risk of endometrial cancer (RR = 0.70, 95% CI: 0.63, 0.78; *I*^2^ = 0%, *p* = 0.60) was found for postmenopausal women, but not for premenopausal women (RR = 0.76 95% CI: 0.49, 1.19; *I*^2^ = 16%, *p* = 0.27). No differences were observed between ever-smokers and non-smokers, i.e., in both subgroups the risk was significantly reduced for the highest category of coffee consumption compared to lowest category (RR = 0.78, 95% CI: 0.68, 0.88 for non-smokers, and RR = 0.74, 95% CI: 0.57, 0.98 for ever-smokers, respectively, Table 2). Finally, the analysis of the highest versus the lowest category of coffee consumption according to body mass index (BMI) categories showed a significant decrease in the risk of endometrial cancer among the obese (BMI > 30 kg/m^2^) women (RR = 0.75, 95% CI: 0.63, 0.88).

### 3.3. Dose–Response Meta-Analysis

**Figure 3:**
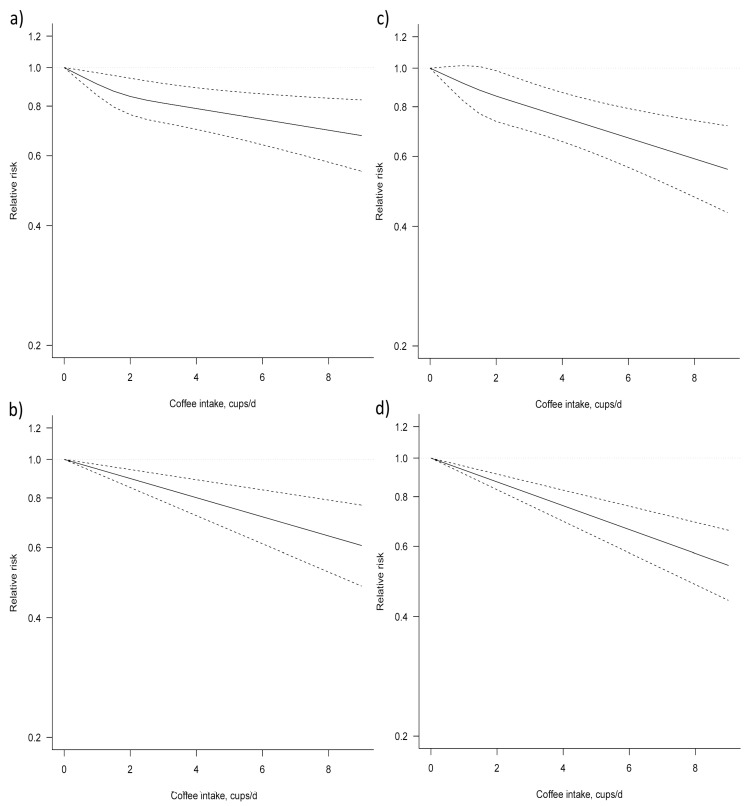
Dose–response association between coffee consumption and endometrial cancer risk (**a**) non-linear, total analysis; (**b**) linear, total analysis (**c**) non-linear, postmenopausal; (**d**) linear, postmenopausal.

Nine studies [16,26,28,29,30,32,34,35,36] were eligible for dose–response meta-analysis of prospective cohort studies on coffee consumption and endometrial cancer risk. Six studies [26,27,28,29,30,31] provided risk estimates for postmenopausal women only. In both the non-linear and the linear dose–response meta-analyses, a significant association between coffee consumption and endometrial cancer risk was found (Figure 3, Table 3). Compared to no coffee consumption, the pooled relative risks for endometrial cancer were: 0.95, 95% CI: 0.92, 0.97, for one cup/day; 0.90, 95% CI: 0.85, 0.94, for two cups/day; 0.85, 95% CI: 0.78, 0.92, for three cups/day; 0.80, 95% CI: 0.72, 0.89, for four cups/day; 0.76, 95% CI: 0.67, 0.86, for five cups/day; 0.72, 95% CI: 0.61, 0.84, for six cups/day; 0.68, 95% CI: 0.57, 0.81, for seven cups/day. Two cohorts, NOWAC and VIP, were excluded from the main analysis, as part of theirs cases are included in the multicentre study EPIC. However, an alternative analysis was performed by including these cohorts and excluding the EPIC study, confirming the results of the main analysis (Supplementary Table S3). Finally, the association between coffee intake and endometrial cancer was stronger when taking into consideration postmenopausal women.

## 4. Discussion

The present meta-analysis, including 12 prospective cohort studies, showed that coffee consumption is associated with a lower risk of endometrial cancer; such association was stronger for postmenopausal endometrial cancer and in obese women (BMI > 30).

Various meta-analyses on the association of coffee consumption and endometrial cancer risk have been conducted so far: for instance, in 2011 Je and Giovannucci [14] documented an inverse dose–response association, with a geographical gradient (stronger association in Japan, followed by the USA and then Europe). Subgroup analyses were later published in 2015 by Yang and colleagues [16] and by Zhou and colleagues [15]: both groups limited their analyses to prospective studies and found a linear inverse association. The first research group found some evidence of heterogeneity among BMI subgroups (overweight women, with a BMI higher than 25, showed a more pronounced inverse association than women with a lower BMI). The second group confirmed these results and pointed out a similar pattern for women without a history of hormone therapy, while documenting no differences with respect to study location, smoking status, and menopausal status. Compared to the previous literature, our results, especially on subgroup analyses for postmenopausal status and BMI (overweight vs obese), are in line with the reported observations, yet provide original findings.

Coffee constituents have been associated with several biological mechanisms related to carcinogenesis, both in vitro and in vivo. These mechanisms include: DNA methylation, oxidative damage, activation of proto-oncogenes and inactivation of onco-suppressor genes, loss of apoptosis and growth control, and induction of angiogenesis [37,38]. Active coffee constituents that have been identified include not only caffeine (mainly known for its ability to increase blood pressure and for its psychostimulatory and diuretic properties) [39], but also polyphenols (e.g., chlorogenic acids, which produce catechins, caffeic, ferulic and coumaric acids), lipids in the form of diterpenes (e.g., cafestol and kahweol), melanoidins, and trigonelline [40,41]. There is evidence that dietary polyphenols might be associated with decreased mortality and cancer risk, and may be the mediators of the potential effects of coffee on cancer prevention [42,43].

With particular referral to female reproductive cancers, several mechanisms have been proposed: for instance, caffeine and coffee intake have been positively associated to sex hormone-binding globulin (SHBG) in postmenopausal women [44]. SHBG is the major carrier of estrogens and testosterone, thus lowering the circulating levels of free hormones; the positive relationship between coffee or caffeine intake and SHBG has been proved in many studies [45,46]. Another possible mechanism resulting in lower levels of circulating estrogens after coffee intake is through the inhibition of the enzyme converting androgens into estrogens, i.e., CYP19 or aromatase [44]. A low level of estrogens is considered a protective factor against endometrial cancers acting through the down-regulation of endometrial proliferation [45], and the inverse association between coffee or caffeine consumption and estrogens has been widely documented [47,48].

Additional effects of coffee and caffeine intake on hormonal functions have been seen in improved insulin sensitivity as a result of the stimulation of insulin-mediated uptake of glucose [49]. Coffee could therefore have a protective role against type 2 diabetes development: most epidemiological studies have documented an inverse association between the intake of caffeinated coffee, decaffeinated coffee, and caffeine and type 2 diabetes in a dose–response manner compared with no or infrequent coffee consumption [50]. In turn, type 2 diabetes has been associated with an increased risk of endometrial cancer, and more specifically with an increased risk of type 1 endometrioid endometrial adenocarcinoma [51]. In recent epidemiological studies, such risk has been associated not only with insulin resistance and diabetes, but also with the metabolic syndrome that is characterized by the coexistence of various factors, such as abdominal obesity, low levels of high density lipoprotein, elevated levels of triglycerides and low levels of density lipoprotein, hypertension, and insulin resistance [52,53].

As already pointed out, we observed a stronger association between coffee consumption and endometrial cancer in women with high BMI (above 30) compared with women with a BMI of 25 or lower. Overweight and obesity have been associated with the development of cancers [54], and various mechanisms have been proposed, including: (i) chronic inflammation and oxidative stress; (ii) cross-talk between tumor cells and surrounding adipocytes; (iii) migration of adipose stromal cells; (iv) obesity-induced hypoxia; (v) genetic susceptibility; and (vi) immunological dysfunctions [55]. In several studies, coffee intake has been inversely associated with metabolic syndrome [56,57,58,59,60,61,62,63,64,65,66]. Metabolic risk factors, such as obesity, impaired glucose tolerance, dyslipidemia, and hypertension have been linked to elevated systemic inflammation and oxidative stress. Thus, impaired metabolism may induce inflammation and oxidative stress, which in turn may lead to carcinogenic transformation. Within these pathways, four main components have been identified: insulin, insulin-like growth factor-I, sex steroids, and adipokines, and coffee consumption has been associated, directly or indirectly, with most of them [55]. For instance, an in vitro study showed that exposure to coffee reduced the accumulation of lipids inhibiting adipocytic differentiation [67]. In animal models, coffee consumption has been related to changes in transcription factors and lipogenesis-related proteins, and, in epidemiological studies, decreased body weight and decreased visceral fat, in relation to coffee consumption, have been observed [68].

The present meta-analysis has some limitations. First, despite we provided insights on underrated factors potentially affecting the association between coffee consumption and endometrial cancer risk, other variables (such as the type of coffee seeds, the roasting method, and the type of preparation) remained largely unexplored. Second, we cannot rule out the possibility of changes in dietary habits (i.e., increased or decreased consumption of coffee) over time, leading to the risk of reverse causation in the event that an individual changed coffee intake due to a newly diagnosed medical condition.

## 5. Conclusions

In conclusions, our findings suggest that increased coffee consumption is associated with decreased risk of endometrial cancer, especially in postmenopausal, obese women.

## Figures and Tables

**Table 1:** Characteristics of the studies included in the meta-analysis.

Author, Year	Cohort Name, Country	Years of Study, Follow-Up	Cases; Total Population	Age Range	Adjustments
Shimazu, 2008	JPHC, Japan	1990–2005, 15 years (maximum)	117; 53,724	40–59 years	Age, study area, BMI (body mass index), menopausal status, age at menopause for postmenopausal women, parity, use of exogenous female hormones, smoking status, green vegetable consumption, beef consumption, pork consumption, and green tea consumption.
Friberg, 2009	SMC, Sweden	1992–2007, 17.6 years (mean)	677; 60,634	40–76 years	Age, BMI, smoking.
Nilsson, 2010	VIP, Sweden	1985–2007, 6 years (median)	108; 32,178	30–60 years	Age, sex, BMI, smoking, education, recreational physical activity.
Giri, 2011	WHI, USA	1993–2005, 7.5 years (average)	427; 45,696	50–79 years	Age, ethnicity, unopposed estrogen use, progestin + estrogen use, smoking, BMI.
Je, 2011	NHS, USA	1980–2006, 26 years (maximum)	672; 67,470	34–59 years	Age, BMI, age at menopause, age at menarche, parity and age at last birth, duration of oral contraceptive use, postmenopausal hormone use, pack-years of smoking, alcohol intake, and total energy intake, tea analysis.
Gunter, 2011	NIH-AARP, USA	1995–2006, 9.3 years (mean)	1486; 111,429	50–71 years	Age, smoking, BMI, age at menarche, age at first child’s birth, parity, age at menopause, HT (hormonal therapy) use, oral contraceptive use, diabetes and physical activity.
Ucella, 2013	IWHS, USA	1986–2005, 20 years (maximum)	542; 23,356	55–69 years	Age, diabetes, duration of HT use, hypertension, age at menarche, age at menopause, BMI, waist-to-hip ratio, smoking status, pack years of smoking, total energy and alcohol use.
Gavrilyuk, 2011	NOWAC, Norway	1991–2007, 10.9 (average)	462; 97,926	30–70 years	Parity, smoking status, BMI, duration of OC (oral contracception) and HRT use.
Weiderpass, 2014	WLH, Sweden	1991–2009, 18 years (maximum)	144; 42,270	30-49 years	Age, education, duration of hormonal contraceptive use, parity, duration of breastfeeding, smoking status and number of cigarettes/day, menopausal status, BMI, and diabetes mellitus.
Merritt, 2015	EPIC, Multicentre; NHS/NHSII USA	EPIC 1992-NA, 11 years (mean); NHS 1976–2010, 25 years (mean); NHSII 1989–2011, 25 years (mean)	EPIC 1303; 301,107; NHS/NHSII 1531; 155,406	EPIC 25–70 years; NHS 30–55 years; NHSII 25–42 years	BMI, total energy intake, smoking status, age at menarche, oral contraceptive use, a combined variable for menopausal status and postmenopausal hormone (PMH) use, parity, and was stratified by the age of recruitment, and the study centre.
Owen Yang, 2015	MWS, UK	1996–2001, 9.3 years (average)	4067; 560,356	~60 years (mean)	Age, region, socioeconomic status, height, age at menarche, parity, duration of oral contraceptive use, age and status of menopause at study baseline, duration of hormone therapy for menopause, BMI, smoking, alcohol consumption, strenuous exercise, tea consumption, and other nonalcohol fluid intake.
Hashibe, 2015	PLCO, USA	1992–2011, 13 years (maximum)	254; 50,563	55–74 years	Age, sex, race, education, smoking status, smoking frequency, smoking duration, time since stopping smoking for past smokers, and drinking frequency.

Abbreviations: EPIC (*European Prospective Investigation into Cancer and Nutrition*); IWHS (Iowa Women’s Health Study); JPHC (Japan Public Health Center-based Prospective Study); NHS (The Nurses’ Health Study); MWS (Million Women Study); NIH-AARP (NIH-AARP Diet and Health Study); NOWAC (The Norwegian Women and Cancer study); PLCO (*Prostate, Lung, Colorectal, and Ovarian Cancer Screening Trial*); SMC (*The Swedish Mammography Cohort*); WHI (Women’s Health Initiative); WLH (Women’s Lifestyle and Health); VIP (Västerbotten intervention project cohort).

**Table 2:** Subgroups and additional analyses of studies reporting the risk of endometrial cancer for the highest versus the lowest (reference) category of coffee consumption (analyses based on 12 studies consisting of 10 databases).

Subgroup/Additional Analysis	No. of Datasets	RR (95% CI)	*I*^2^	*P_heterogeneity_*
Total	10	0.79 (0.73, 0.87)	28%	0.19
Geographical area				
North America	5	0.75 (0.67, 0.84)	6%	0.37
Europe	4	0.84 (0.74, 0.94)	29%	0.24
Asia	1	0.97 (0.56, 1.68)	NA	NA
Menopausal status				
Postmenopausal	7	0.70 (0.63, 0.78)	0%	0.60
Premenopausal	2	0.76 (0.49, 1.19)	16%	0.27
Coffee type				
Caffeinated	4	0.65 (0.50, 0.85)	64%	0.04
Decaffeinated	4	0.76 (0.62, 0.93)	0%	0.72
BMI				
<25 kg/m^2^	7	0.99 (0.86, 1.14)	0%	0.58
>25 kg/m^2^	7	0.79 (0.61, 1.01)	66%	0.004
>30 kg/m^2^	5	0.75 (0.63, 0.88)	22%	0.27
Smoking status				
Never smoker	8	0.78 (0.68, 0.88)	7%	0.38
Ever smoker (former/current)	8	0.74 (0.57, 0.98)	68%	0.003
Adjusted for smoking				
No	0	NA	NA	NA
Yes	10	0.79 (0.73, 0.87)	28%	0.19
Adjusted for BMI				
No	1	0.69 (0.52, 0.91)	NA	NA
Yes	9	0.80 (0.74, 0.88)	27%	0.20
Adjusted for education				
No	8	0.81 (0.74, 0.89)	30%	0.19
Yes	2	0.68 (0.53, 0.87)	0%	0.80
Adjusted for alcohol intake				
No	8	0.77 (0.71, 0.84)	0%	0.56
Yes	2	0.85 (0.67, 1.07)	52%	0.15

**Table 3:** Dose–response meta-analysis of prospective cohort studies on coffee consumption and endometrial cancer risk.

	No. of Datasets (No. of Studies)	Coffee Intake (Cups/Day)								*I*^2^*(%)*	*P_heterogeneity_*	*P_non-linearity_*
		0	1	2	3	4	5	6	7			
Total analysis												
Non-linear	11 (9)	Ref.	0.91 (0.85, 0.97)	0.85 (0.76, 0.94)	0.81 (0.73, 0.91)	0.79 (0.70, 0.89)	0.76 (0.67, 0.87)	0.74 (0.64, 0.86)	0.72 (0.61, 0.85)	30.98	0.09	0.09
Linear	11 (9)	Ref.	0.95 (0.92, 0.97)	0.90 (0.85, 0.94)	0.85 (0.78, 0.92)	0.80 (0.72, 0.89)	0.76 (0.67, 0.86)	0.72 (0.61, 0.84)	0.68 (0.57, 0.81)	59.21	0.01	NA
Postmenopausal												
Non-linear	7 (6)	Ref.	0.92 (0.83, 1.01)	0.85 (0.73, 0.99)	0.80 (0.69, 0.92)	0.75 (0.65, 0.87)	0.71 (0.61, 0.83)	0.67 (0.56, 0.79)	0.63 (0.52, 0.76)	0	0.64	0.67
Linear	7 (6)	Ref.	0.93 (0.91, 0.95)	0.87 (0.83, 0.91)	0.81 (0.76, 0.87)	0.76 (0.69, 0.83)	0.71 (0.63, 0.79)	0.66 (0.58, 0.76)	0.62 (0.53, 0.72)	0	0.46	NA

## Supplementary Materials

The following are available online at www.mdpi.com/2072-6643/9/11/1223/s1.

## References

[1] Word Health Oranganization. http://globocan.iarc.fr/Default.aspx.

[2] Forouzanfar M.H., Alexander L., Anderson H.R., Bachman V.F., Biryukov S., Brauer M., Burneet R., Casey D., Coates M.M., Cohen A. (2016). Global, regional, and national comparative risk assessment of 79 behavioural, environmental and occupational, and metabolic risks or clusters of risks, 1990–2013: A systematic analysis for the global burden of disease study 2013. Lancet.

[3] Grosso G., Bella F., Godos J., Sciacca S., Del Rio D., Ray S., Galvano F., Giovannucci E. (2017). Possible role of diet in cancer: Systematic review and multiple meta-analyses of dietary patterns, lifestyle factors, and cancer risk. Nut. Rev..

[4] Lim S.S., Vos T., Flaxman A.D., Danaei G., Shibuya K., Adair-Rohani H., AlMazroa M.A., Amann M., Anderson H.R., Andrews K.G. (2012). A comparative risk assessment of burden of disease and injury attributable to 67 risk factors and risk factor clusters in 21 regions, 1990–2010: A systematic analysis for the global burden of disease study 2010. Lancet.

[5] Grosso G., Godos J., Galvano F., Giovannucci E.L. (2017). Coffee, caffeine, and health outcomes: An umbrella review. Annu. Rev. Nutr..

[6] Grosso G., Micek A., Godos J., Sciacca S., Pajak A., Martinez-Gonzalez M.A., Giovannucci E.L., Galvano F. (2016). Coffee consumption and risk of all-cause, cardiovascular, and cancer mortality in smokers and non-smokers: A dose-response meta-analysis. Eur. J. Epidemiol..

[7] Marventano S., Salomone F., Godos J., Pluchinotta F., Del Rio D., Mistretta A., Grosso G. (2016). Coffee and tea consumption in relation with non-alcoholic fatty liver and metabolic syndrome: A systematic review and meta-analysis of observational studies. Clin. Nutr..

[8] Godos J., Micek A., Marranzano M., Salomone F., Rio D.D., Ray S. (2017). Coffee consumption and risk of biliary tract cancers and liver cancer: A dose-response meta-analysis of prospective cohort studies. Nutrition.

[9] Liu H., Hu G.H., Wang X.C., Huang T.B., Xu L., Lai P., Guo Z.F., Xu Y.F. (2015). Coffee consumption and prostate cancer risk: A meta-analysis of cohort studies. Nutr. Cancer.

[10] Ran H.Q., Wang J.Z., Sun C.Q. (2016). Coffee consumption and pancreatic cancer risk: An update meta-analysis of cohort studies. Pak. J. Med. Sci..

[11] Gan Y., Wu J., Zhang S., Li L., Cao S., Mkandawire N., Ji K., Herath C., Gao C., Xu H. (2016). Association of coffee consumption with risk of colorectal cancer: A meta-analysis of prospective cohort studies. Oncotarget.

[12] Caprioli G., Cortese M., Sagratini G., Vittori S. (2015). The influence of different types of preparation (espresso and brew) on coffee aroma and main bioactive constituents. Int. J. Food Sci. Nutr..

[13] Godos J., Pluchinotta F.R., Marventano S., Buscemi S., Li Volti G., Galvano F., Grosso G. (2014). Coffee components and cardiovascular risk: Beneficial and detrimental effects. Int. J. Food Sci. Nutr..

[14] Je Y., Giovannucci E. (2012). Coffee consumption and risk of endometrial cancer: Findings from a large up-to-date meta-analysis. Int. J. Cancer.

[15] Zhou Q., Luo M.L., Li H., Li M., Zhou J.G. (2015). Coffee consumption and risk of endometrial cancer: A dose-response meta-analysis of prospective cohort studies. Sci. Rep..

[16] Yang T.O., Crowe F., Cairns B.J., Reeves G.K., Beral V. (2015). Tea and coffee and risk of endometrial cancer: Cohort study and meta-analysis. Am. J. Clin. Nutr..

[17] Federation E.C. Coffee Consumption in Europe. https://www.ecf-coffee.org/index.php.

[18] Grigg D. (2002). The worlds of tea and coffee: Patterns of consumption. GeoJournal.

[19] Wang A., Wang S., Zhu C., Huang H., Wu L., Wan X., Yang X., Zhang H., Miao R., He L. (2016). Coffee and cancer risk: A meta-analysis of prospective observational studies. Sci. Rep..

[20] Stroup D.F., Berlin J.A., Morton S.C., Olkin I., Williamson G.D., Rennie D., Moher D., Becker B.J., Sipe T.A., Thacker S.B. (2000). Meta-analysis of observational studies in epidemiology: A proposal for reporting. Meta-analysis of observational studies in epidemiology (moose) group. JAMA.

[21] Wells G.A., Shea B., O’Connell D., Peterson J., Welch V., Losos M., Tugwell P. (1999). The Newcastle-Ottawa Scale (nos) for Assessing the Quality of Nonrandomised Studies in Meta-Analyses.

[22] Greenland S. (1987). Quantitative methods in the review of epidemiologic literature. Epidemiol. Rev..

[23] Greenland S., Longnecker M.P. (1992). Methods for trend estimation from summarized dose-response data, with applications to meta-analysis. Am. J. Epidemiol..

[24] Orsini N.B.R., Greenland S. (2006). Generalized least squares for trend estimation of summarized dose-response data. Stata J..

[25] Orsini N., Li R., Wolk A., Khudyakov P., Spiegelman D. (2012). Meta-analysis for linear and nonlinear dose-response relations: Examples, an evaluation of approximations, and software. Am. J. Epidemiol..

[26] Friberg E., Orsini N., Mantzoros C.S., Wolk A. (2009). Coffee drinking and risk of endometrial cancer—A population-based cohort study. Int. J. Cancer.

[27] Gavrilyuk O., Braaten T., Skeie G., Weiderpass E., Dumeaux V., Lund E. (2014). High coffee consumption and different brewing methods in relation to postmenopausal endometrial cancer risk in the Norwegian women and cancer study: A population-based prospective study. BMC Women's Health.

[28] Giri A., Sturgeon S.R., Luisi N., Bertone-Johnson E., Balasubramanian R., Reeves K.W. (2011). Caffeinated coffee, decaffeinated coffee and endometrial cancer risk: A prospective cohort study among us postmenopausal women. Nutrition.

[29] Gunter M.J., Schaub J.A., Xue X., Freedman N.D., Gaudet M.M., Rohan T.E., Hollenbeck A.R., Sinha R. (2012). A prospective investigation of coffee drinking and endometrial cancer incidence. Int. J. Cancer.

[30] Hashibe M., Galeone C., Buys S.S., Gren L., Boffetta P., Zhang Z.F., La Vecchia C. (2015). Coffee, tea, caffeine intake, and the risk of cancer in the plco cohort. Br. J. Cancer.

[31] Je Y., Hankinson S.E., Tworoger S.S., De Vivo I., Giovannucci E. (2011). A prospective cohort study of coffee consumption and risk of endometrial cancer over a 26-year follow-up. Cancer Epidemiol. Biomark. Prev..

[32] Merritt M.A., Tzoulaki I., Tworoger S.S., De Vivo I., Hankinson S.E., Fernandes J., Tsilidis K.K., Weiderpass E., Tjonneland A., Petersen K.E. (2015). Investigation of dietary factors and endometrial cancer risk using a nutrient-wide association study approach in the epic and nurses’ health study (NHS) and NHSII. Cancer Epidemiol. Biomark. Prev..

[33] Nilsson L.M., Johansson I., Lenner P., Lindahl B., Van Guelpen B. (2010). Consumption of filtered and boiled coffee and the risk of incident cancer: A prospective cohort study. Cancer Causes Control.

[34] Shimazu T., Inoue M., Sasazuki S., Iwasaki M., Kurahashi N., Yamaji T., Tsugane S., JPHC Study Group Members of the Japan Public Health Center-based Prospective Study (2008). Coffee consumption and risk of endometrial cancer: A prospective study in Japan. Int. J. Cancer.

[35] Uccella S., Mariani A., Wang A.H., Vierkant R.A., Cliby W.A., Robien K., Anderson K.E., Cerhan J.R. (2013). Intake of coffee, caffeine and other methylxanthines and risk of Type I vs. Type II endometrial cancer. Br. J. Cancer.

[36] Weiderpass E., Sandin S., Lof M., Oh J.K., Inoue M., Shimazu T., Tsugane S., Adami H.O. (2014). Endometrial cancer in relation to coffee, tea, and caffeine consumption: A prospective cohort study among middle-aged women in Sweden. Nutr. Cancer.

[37] Bohn S.K., Blomhoff R., Paur I. (2014). Coffee and cancer risk, epidemiological evidence, and molecular mechanisms. Mol. Nutr. Res..

[38] Boettler U., Sommerfeld K., Volz N., Pahlke G., Teller N., Somoza V., Lang R., Hofmann T., Marko D. (2011). Coffee constituents as modulators of Nrf2 nuclear translocation and ARE (EpRE)-dependent gene expression. J. Nutr. Biochem..

[39] Gaascht F., Dicato M., Diederich M. (2015). Coffee provides a natural multitarget pharmacopeia against the hallmarks of cancer. Genes Nutr..

[40] Niseteo T., Komes D., Belščak-Cvitanović A., Horžić D., Budeč M. (2012). Bioactive composition and antioxidant potential of different commonly consumed coffee brews affected by their preparation technique and milk addition. Food Chem..

[41] Liang N., Kitts D.D. (2014). Antioxidant property of coffee components: Assessment of methods that define mechanisms of action. Molecules.

[42] Grosso G., Godos J., Lamuela-Raventos R., Ray S., Micek A., Pajak A., Sciacca S., D’Orazio N., Del Rio D., Galvano F. (2017). A comprehensive meta-analysis on dietary flavonoid and lignan intake and cancer risk: Level of evidence and limitations. Mol. Nutr. Food Res..

[43] Grosso G., Micek A., Godos J., Pajak A., Sciacca S., Galvano F., Giovannucci E.L. (2017). Dietary flavonoid and lignan intake and mortality in prospective cohort studies: Systematic review and dose-response meta-analysis. Am. J. Epidemiol..

[44] Kotsopoulos J., Eliassen A.H., Missmer S.A., Hankinson S.E., Tworoger S.S. (2009). Relationship between caffeine intake and plasma sex hormone concentrations in premenopausal and postmenopausal women. Cancer.

[45] Ferrini R.L., Barrett-Connor E. (1996). Caffeine intake and endogenous sex steroid levels in postmenopausal women. The rancho bernardo study. Am. J. Epidemiol..

[46] Nagata C., Kabuto M., Shimizu H. (1998). Association of coffee, green tea, and caffeine intakes with serum concentrations of estradiol and sex hormone-binding globulin in premenopausal Japanese women. Nutr. Cancer.

[47] Fung T.T., Schulze M.B., Hu F.B., Hankinson S.E., Holmes M.D. (2012). A dietary pattern derived to correlate with estrogens and risk of postmenopausal breast cancer. Breast Cancer Res. Treat..

[48] Sisti J.S., Hankinson S.E., Caporaso N.E., Gu F., Tamimi R.M., Rosner B., Xu X., Ziegler R., Eliassen A.H. (2015). Caffeine, coffee, and tea intake and urinary estrogens and estrogen metabolites in premenopausal women. Cancer Epidemiol. Biomark. Prev..

[49] Akash M.S., Rehman K., Chen S. (2014). Effects of coffee on type 2 diabetes mellitus. Nutrition.

[50] Ortega Á., Berná G., Rojas A., Martín F., Soria B. (2017). Gene-diet interactions in type 2 diabetes: The chicken and egg debate. Int. J. Mol. Sci..

[51] Lees B., Leath C.A. (2015). The impact of diabetes on gynecologic cancer: Current status and future directions. Curr. Obstet. Gynecol. Rep..

[52] Alicandro G., Tavani A., La Vecchia C. (2017). Coffee and cancer risk: A summary overview. Eur. J. Cancer Prev..

[53] Stocks T., Bjorge T., Ulmer H., Manjer J., Haggstrom C., Nagel G., Engeland A., Johansen D., Hallmans G., Selmer R. (2015). Metabolic risk score and cancer risk: Pooled analysis of seven cohorts. Int. J. Epidemiol..

[54] Renehan A.G., Tyson M., Egger M., Heller R.F., Zwahlen M. (2008). Body-mass index and incidence of cancer: A systematic review and meta-analysis of prospective observational studies. Lancet.

[55] De Pergola G., Silvestris F. (2013). Obesity as a major risk factor for cancer. J. Obes..

[56] Dos Santos P.R., Ferrari G.S., Ferrari C.K. (2015). Diet, sleep and metabolic syndrome among a legal amazon population, Brazil. Clin. Nutr. Res..

[57] Driessen M.T., Koppes L.L., Veldhuis L., Samoocha D., Twisk J.W. (2009). Coffee consumption is not related to the metabolic syndrome at the age of 36 years: The amsterdam growth and health longitudinal study. Eur. J. Clin. Nutr..

[58] Chang C.S., Chang Y.F., Liu P.Y., Chen C.Y., Tsai Y.S., Wu C.H. (2012). Smoking, habitual tea drinking and metabolic syndrome in elderly men living in rural community: The tianliao old people (TOP) study 02. PLoS ONE.

[59] Grosso G., Marventano S., Galvano F., Pajak A., Mistretta A. (2014). Factors associated with metabolic syndrome in a mediterranean population: Role of caffeinated beverages. J. Epidemiol..

[60] Grosso G., Stepaniak U., Micek A., Topor-Madry R., Pikhart H., Szafraniec K., Pajak A. (2015). Association of daily coffee and tea consumption and metabolic syndrome: Results from the polish arm of the hapiee study. Eur. J. Nutr..

[61] Lutsey P.L., Steffen L.M., Stevens J. (2008). Dietary intake and the development of the metabolic syndrome: The atherosclerosis risk in communities studies. Circulation.

[62] Matsuura H., Mure K., Nishio N., Kitano N., Nagai N., Takeshita T. (2012). Relationship between coffee consumption and prevalence of metabolic syndrome among Japanese civil servants. J. Epidemiol..

[63] Nordestgaard A.T., Thomsen M., Nordestgaard B.G. (2015). Coffee intake and risk of obesity, metabolic syndrome and type 2 diabetes: A Mendelian randomization study. Int. J. Epidemiol..

[64] Takami H., Nakamoto M., Uemura H., Katsuura S., Yamaguchi M., Hiyoshi M., Sawachika F., Juta T., Arisawa K. (2013). Inverse correlation between coffee consumption and prevalence of metabolic syndrome: Baseline survey of the japan multi-institutional collaborative cohort (J-micc) study in Tokushima, Japan. J. Epidemiol..

[65] Suliga E., Koziel D., Ciesla E., Rebak D., Gluszek S. (2016). Coffee consumption and the occurrence and intensity of metabolic syndrome: A cross-sectional study. Int. J. Sci. Nutr..

[66] Micek A., Grosso G., Polak M., Kozakiewicz K., Tykarski A., Puch Walczak A., Drygas W., Kwasniewska M., Pajak A. (2017). Association between tea and coffee consumption and prevalence of metabolic syndrome in Poland—Results from the WOBASZ II study (2013–2014). Int. J. Food Sci. Nutr..

[67] Aoyagi R., Funakoshi-Tago M., Fujiwara Y., Tamura H. (2014). Coffee inhibits adipocyte differentiation via inactivation of pparγ. Biol. Pharm. Bull..

[68] Kearney J.M., Kearney M.J., McElhone S., Gibney M.J. (1999). Methods used to conduct the pan-European Union survey on consumer attitudes to physical activity, body weight and health. Public Health Nutr..

